# Variation in restraint use between hospitals: a multilevel analysis of multicentre prevalence measurements in Switzerland and Austria

**DOI:** 10.1186/s12913-021-06362-y

**Published:** 2021-04-20

**Authors:** Silvia Thomann, Sabine Hahn, Silvia Bauer, Dirk Richter, Sandra Zwakhalen

**Affiliations:** 1 Department of Health Professions, Applied Research & Development in Nursing, Bern University of Applied Sciences, Murtenstrasse 10, 3008 Bern, Switzerland; 2 Department of Nursing Science, Medical University of Graz, Universitätsplatz 4, 8010 Graz, Austria; 3 Center for Psychiatric Rehabilitation, Bern University Hospital for Mental Health, Murtenstrasse 46, 3008 Bern, Switzerland; 4 University Hospital for Psychiatry and Psychotherapy, University of Bern, Bolligenstrasse 111, 3060 Bern, Switzerland; 5 Department of Health Services Research, Care and Public Health Research Institute, Maastricht University, PO BOX 616, 6200 MD Maastricht, The Netherlands

**Keywords:** Hospitals, Multilevel analysis, Organisational culture, Quality of health care, Restraint

## Abstract

**Background:**

In restraint use in the somatic acute-care hospital setting, routine and institutional culture seem to play an important role. This implies that similar patient situations would be managed with restraints in one hospital, while in another hospital the situation would be managed without restraints. This practice variation appears to be ethically and legally questionable. The influence of organisation-specific factors such as the availability of guidelines is discussed. However, the relevance of such factors at the hospital level has been rarely investigated to date. Therefore, the aims of this study were a) to determine how much variance in restraint use can be explained on the hospital level (hospital general effect) and b) to examine the impact of organisational factors on restraint use (specific contextual effects).

**Methods:**

A secondary data analysis of cross-sectional multicentre data was performed. Data were collected during three quality measurements (2016–2018) in acute-care hospitals in Switzerland and Austria. Hospitalised patients from different medical specialties aged 18+ with informed consent were included. Descriptive analysis and multilevel logistic regression analysis were performed.

**Results:**

The study included 29,477 patients from a total of 140 hospitals. The 30-day prevalence rate of patients with at least one restraint was 8.7% (*n* = 2577). The availability of guidelines regarding restraint use and refresher courses for nursing staff were associated with less restraint use (odds ratios = 0.60 and 0.75). By adding the hospital as a random effect, the explained variance of the model increased from 24 to 55%.

**Conclusions:**

The use of restraints varies widely between hospitals, even considering patient characteristics. The identification of situations in which restraints were used out of routine or institutional culture appears to be an important approach in restraint reduction. Investments in appropriate structures and employee knowledge can facilitate providing restraint-free care as much as possible.

**Supplementary Information:**

The online version contains supplementary material available at 10.1186/s12913-021-06362-y.

## Background

Restraint use in health care often leads to negative effects for patient health, such as functional decline, higher mortality, distress or trauma [1–4], and to moral distress for health professionals [5, 6]. Therefore, a reduction in restraint use is recommended [7–9].

To date, quality improvement initiatives regarding restraint use are mainly known in the long-term care and mental health setting [10, 11]. Nevertheless, restraints are frequently used in the somatic acute care hospital setting (henceforth referred to as ‘hospital’) as well. Prevalence rates up to 100% are reported [1, 12, 13]. Large differences in restraint prevalence rates can be detected depending on the ward type studied (intensive care units often have a much higher prevalence rate) and by the definition of restraints used (e.g. only restraint belts; alternatively, bed rails and electronic monitoring can also be considered as restraints).

Frequently stated reasons for restraint use in the hospital setting are patient safety (e.g. fall prevention or prevention of therapy interruption) and patient characteristics like cognitive impairment [5, 14, 15]. However, evidence for the effectiveness of restraints for these reasons is lacking [5, 16, 17]. Nevertheless, restraints also seem to be used out of routine according to the tradition on the ward or local habits [18–21]. This implies that practice variation may exist. Consequently, in a similar patient situation, restraints may be used in one hospital, while in another hospital this situation would be managed without restraints. These differences in restraint use among hospitals independent of evidence or professional recommendations appear to be ethically and legally questionable. In this context, the relevance and role of organisational factors such as structures, policies/guidelines, education for staff, monitoring of restraint use and organisational attitudes are discussed [18, 20–24].

Surprisingly, the practice variation in restraint use among hospitals (hospital general effect) and the impact of organisational factors (specific contextual effects) has rarely been investigated to date. Nevertheless, in order to promote a professional management of restraints and, thus, to develop and implement effective measures for restraint reduction, it is crucial to know the influencing factors on different levels and their impact on the use and non-use of restraints. Therefore, the aims of this study were a) to determine how much variance in restraint use can be explained on the hospital level (hospital general effect) and b) to examine the impact of organisational factors (specific contextual effects) on restraint use; both aspects considered the influence of patient characteristics on restraint use.

## Methods

### Study design and setting

A secondary data analysis of cross-sectional multicentre studies was performed. Data were collected within the International Prevalence Measurement of Quality of Care, called LPZ (Landelijke Prevalentiemeting Zorgkwaliteit) International [25, 26]. LPZ International performs an annual international quality measurement for a variety of care indicators (like pressure ulcers, falls and restraints) in various settings and countries. Healthcare institutions are invited annually by a national coordinator in several countries to participate on a voluntary basis in the measurement. For the present study, data from the hospital setting of Switzerland and Austria from three one-day measurement points in the years 2016 to 2018 were included. Other countries in the LPZ consortium were not able to provide data as very few hospitals measured restraint use.

### Sample

In the LPZ measurement, hospitalised patients from different medical specialties (ward types) aged 18+ with informed verbal (Switzerland) or written (Austria) consent were included. Patients were excluded from the LPZ measurement if they were not available on the ward during the measurement (e.g. since they were undergoing surgery) or could not give informed consent (e.g. due to cognitive impairment or language barriers) and where no legal representative was available. There were no additional exclusion criteria for this secondary analysis.

### Instrument and data collection

For data collection, the LPZ 2.0 instrument was used. It is the 2016 revised version of the LPZ instrument [25]. With LPZ 2.0, general and care indicator specific information is assessed on the institutional, ward and patient levels. For this secondary data analysis, information regarding restraints of different levels was included (for details, see Table 1). Restraints were defined as ‘interventions that may infringe [on] a person’s human rights and freedom of movement, including observation, seclusion, manual restraint, mechanical restraint and rapid tranquillisation’ [27].

**Table 1:** Variables

Level	Information
National	Country (Switzerland, Austria)
Institutional	Availability of a protocol/guidelines regarding restraints (based on a(n) (inter) national guideline) within the institution (yes, no)
	Availability of a multi-disciplinary expert committee regrading restraints within the institution (yes, no)
Ward	Regular audits are performed on the ward level to ensure compliance with the protocol/guidelines regarding restraints (yes, no)
	Refresher course regarding restraints for at least 80% of ward nursing staff in the last 2 years (yes, no)
Patient	Age in years (interval)
	Female gender (yes, no)
	Surgical intervention in the 2 weeks prior to data collection (yes, no)
	Number of days since admission to hospital (interval)
	Medical diagnosis groups according to International Statistical Classification of Diseases and Related Health Problems 10^th^ Revision (ICD-10; for each diagnosis group yes, no) [28]
	Care dependency assessed with the Care Dependency Scale (CDS) (15 items [e.g., eating and drinking or mobility] are rated on a Likert scale from 1 to 5 [sum score 15–75]. Lower scores indicate higher care dependency resulting in five verified categories: 15–24 completely dependent, 25–44 dependent to a great extent, 45–59 partially dependent, 60–69 independent to a great extent, 70–75 completely independent) [29]
	Restraint use within the institution retrospectively over a maximum period of 30 days (yes, no)

Within LPZ 2.0, the data collection process is highly standardised. The whole process (e.g. recruitment and information of patients, preparing data collection including documentation of restraint use 30 days prior to data collection) and all questions and answer options are internationally defined and described in a measurement manual. Additionally, the questionnaire was conceived as an online data entry program leading the questionnaire completion. To ensure uniform execution of the measurement and uniform answering of the questions, data collectors were trained. Using the train-the-trainer procedure, the national coordinator trained the responsible person within each hospital (called the institutional coordinator). The institutional coordinator then trained the data collectors (registered nurses) within the hospital. Additionally, the measurement manual with all the information was made available for the data collectors directly in the data entry program.

On the predetermined measurement day, the patient level data were collected by the trained data collectors on-site at the patient’s bedside and/or through patient documentation (retrospective assessment). The questions on the institutional and ward level were answered by the institutional coordinator.

### Statistical analysis

The data from the different measurement points and the two countries (Switzerland and Austria) were pooled into one dataset. Descriptive statistics (numbers, percentages, 95% confidence intervals [CI], median, interquartile range [IQR]) were used to describe the organisational factors, the sample and the restraint prevalence rate.

**Fig. 1:**
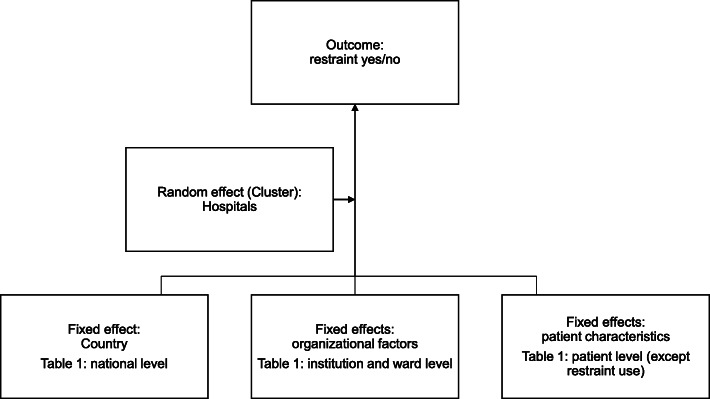
Baseline multilevel regression model

A multilevel modelling approach was used in order to determine how much variance in restraint use can be explained on the hospital level (hospital general effect). This means that the analysis took into account that patients are clustered in hospitals with their organisational factors. Such methodological approaches are particularly well known from public health research where, for example, the influence of neighbourhoods on certain behaviours is studied [30, 31]. The baseline (before variable selection) of the multilevel logistic regression model of our study was built as shown in Fig. 1. We could not include the ward level due to patient transfers between wards and potential misclassification as the exact ward where the restraint has happened was not recorded during data collection.

Due to the limited data on restraint use in the hospital setting (with partial exception of the ICU), designing a purely theory-based model was not possible, respectively, the insufficient theoretical basis entailed the risk of inaccurate assumptions for including or excluding patient level data. Given that the “blind” inclusion of all possible fixed effects carries the risk of overadjustment, we decided on a data-driven model with variable selection (explorative design).

For variable selection, we used the Akaike information criterion (AIC) [32] backwards procedure implemented in the R package MASS [33]. Here, however, the hospital random effect had to be treated as a fixed effect. During development of the analysis, we also considered using similar variable selection procedures for logistic multilevel models, but the few software implementations we found were not practicable for our problem. Since the hospital general effect is an explicit part of the question, the AIC procedure was employed such that the hospital variable cannot be unselected. Further, to enhance the stability of the variable selection, i.e. to reduce the number of noisy variables selected due to the large sample size, we used a split-half approach where the AIC procedure was applied on both of two subsets from a random split of the data, and then only the variables included in both selections were used for the final multilevel model. The model then was built as a generalised linear mixed model fit by maximum likelihood (Laplace approximation) implemented in the R package Ime4 [34]. The ICC (intraclass correlation coefficient) was estimated, and a log-likelihood ratio test was performed to evaluate the relevance of the random effect. However, as the ICC is difficult to interpret for logistic multilevel models we calculated the median odds ratio (MOR) of the random effect [30, 31]. The MOR allows to translate the hospital level variance into the same scale as the fixed effects are reported (OR). In addition, the 80% interval odds ratio (IOR) was calculated for the organisational factors (specific contextual effects) included in the fixed effects. Using the 80% IOR it can be better considered that these characteristics can take on only one value per hospital (cluster) [31]. These two calculations were performed using the calculation sheet provided by Merlo, Wagner [30]. The R codes of the multilevel analysis are available in the Additional File 1.Three ICD-10 diagnosis groups (congenital malformations, deformations and chromosomal abnormalities; certain conditions originating in the perinatal period; pregnancy, childbirth and the puerperium) and the answer option unknown/no diagnosis were present in less than 1% of patients and would have led to convergence problems of the regression model. Therefore, these variables had to be excluded. For similar reasons, the variables Age in years and Number of days since admission to hospital had to be standardised. Since there is a non-linear association of age and restraint use the variable Age in years was also included as quadratic (squared) term (second-order polynomial) in the multilevel model. Multicollinearity was tested using the variance inflation factor (VIF). There were no missing data as the online data entry program only allowed for finishing the survey when all questions were answered.

The statistical analysis was conducted utilising R Version 4.0.1 [35] and the R Packages compareGroups [36], Hmisc [37], Ime4 [34], jtools [38], MASS [33], MuMIn [39], sjPlot [40], tableone [41] and tidyverse [42]. For data cleaning and pooling, SPSS version 25 was used [43].

### Ethical considerations

In Switzerland, the Ethics Committee of the Canton of Bern declared that the present study is not subject to the Swiss Human Research Act and ethical approval was not required (April 2019, BASEC-Nr: Req-2019-00259). In Austria, the Ethics Committee of the Medical University of Graz approved the study protocol (approval nr. 20–192 ex08/09). All patients or their legal representatives received written information about the measurement and gave their verbal (Switzerland) or written (Austria) informed consent. Data were collected pseudonymously so that no conclusions can be made regarding the individual patients. Participation was voluntary.

## Results

The study included 29,477 patients from a total of 1117 wards in 140 hospitals (Table 2). Of these, 20,561 (69.8%) patients were assessed at 84 hospitals in Switzerland and 8916 (30.2%) patients at 56 hospitals in Austria. The number of participating patients per hospital ranged from 2 to 1718 with a median of 102 (Switzerland: range from 2 to 1718, median 146; Austria: range from 16 to 979, median 73). Response rate of all patients hospitalized (*N* = 39,106) on the measurement days in the 140 hospitals was 75.4% (95% confidence interval [CI] 74.9–75.8%; Switzerland: 76.3% [95% CI = 75.8–76.8%] *N* = 26,934; Austria 73.3% [95% CI = 72.5–74.0%] *N* = 12,172).

**Table 2:** Sample description

Characteristics	Total (***n*** = 29,477)		Switzerland (***n*** = 20,561)		Austria (***n*** = 8916)	
**Institutional level**	**n**	**% (95% CI)**	**n**	**% (95% CI)**	**n**	**% (95% CI)**
Guidelines regarding restraint use (yes)	21,694	73.6 (73.1–74.1)	14,318	69.6 (69.0–70.3)	7376	82.7 (81.9–83.5)
Multi-disciplinary expert committee (yes)	12,575	42.7 (42.1–43.2)	7302	35.5 (34.9–36.2)	5273	59.1 (58.1–60.2)
**Ward level**						
Regular audits (yes)	20,126	68.3 (67.7–68.8)	13,893	67.6 (66.9–68.2)	6233	69.9 (68.9–70.9)
Refresher course (yes)	6209	21.1 (20.6–21.5)	2280	11.1 (10.7–11.5)	3929	44.1 (43.0–45.1)
**Patient level**	**median**	**IQR**	**median**	**IQR**	**median**	**IQR**
Age in years	70	24	70	23	69	23
Number of days since admission to hospital	5	9	5	9	5	10
Care Dependency Scale (sum score)	71	15	70	15	74	11
	**n**	**% (95% CI)**	**n**	**% (95% CI)**	**n**	**% (95% CI)**
Female gender	14,504	49.2 (48.6–49.8)	9902	48.2 (47.5–48.8)	4602	51.6 (50.6–52.7)
Surgical intervention in the 2 weeks prior to data collection (yes)	10,542	35.8 (35.2–36.3)	8318	40.5 (39.8–41.1)	2224	24.9 (24.0–25.9)
Three most frequent ICD-10 diagnosis groups (multiple responses)						
Diseases of the circulatory system	16,245	55.1 (54.5–55.7)	11,756	57.2 (56.5–57.9)	4489	50.3 (49.3–51.4)
Endocrine, nutritional and metabolic diseases	9886	33.5 (33.0–34.1)	7023	34.2 (33.5–34.8)	2863	32.1 (31.1–33.1)
Diseases of the musculoskeletal system and connective tissue	9834	33.4 (32.8–33.9)	7543	36.7 (36.0–37.3)	2291	25.7 (24.8–26.6)
Restraint (yes)	2577	8.7 (8.4–9.1)	2171	10.6 (10.1–11.0)	406	4.6 (4.1–5.0)

*IQR* interquartile range, *95% CI* 95% confidence interval, *ICD-10* International Statistical Classification of Diseases and Related Health Problems 10^th^ Revision

The 30-day prevalence rate of patients with at least one restraint was 8.7% (*n* = 2577). Differences between countries were evident. In Switzerland, the prevalence rate was much higher (10.6%, *n* = 2171) than in Austria (4.6%, *n* = 406). A more refined description about the differences on patient level between Switzerland and Austria as well as about the restraint type used, reasons for restraint use and process indicators is available in a publication by Thomann et al. [15].

Overall, 73.6% (*n* = 21,694) of all patients were treated in a hospital with guidelines regarding restraints. A multi-disciplinary expert committee regarding restraints was implemented in the hospitals of 42.7% (*n* = 12,575) of all patients assessed. On the ward level, regular audits to ensure compliance with the guidelines regarding restraints was performed in 68.3% (*n* = 20,126) of all patients surveyed. In 21.1% (*n* = 6209) of all patient situations, nursing staff attended a refresher course on restraints.

Based on the multilevel regression analysis, several factors associated with restraint use were found (Table 3). The strongest association was found for patients’ care dependency: completely dependent patients in comparison to completely independent patients had an almost 40 times higher risk of being restrained (odds ratio [OR] 39.74, 95% confidence interval [CI] 32.72–48.26). A strong association was also found for patients with mental and behavioural disorders: the risk for them to be restrained was more than two times higher than for patients without such disorders (OR 2.31, 95% CI 2.09–2.56).

**Table 3:** Multilevel logistic regression model

Model: AIC 12568.9; marginal *R*^2^ = 0.24; conditional *R*^2^ = 0.55; ICC = 0.41; MOR = 4.22	
**Random effect**	**Variance (SD)**
Hospital (intercept)	2.28 (1.51)
**Fixed effects**	**OR (95% CI)**
(intercept)	0.02 (0.01–0.03)^a^
*Organisational factors (specific contextual effects)*	
Guidelines regarding restraint (yes)	0.60 (0.49–0.74)^a^
	80% IOR: (0.04–9.30)
Refresher course regarding restraints (yes)	0.75 (0.64–0.89)^a^
	80% IOR: (0.05–11.64)
*Patient characteristics*	
Age in years (1^st^ degree)	1.21 (1.14–1.29)^a^
Age in years squared (2^nd^ degree)	1.11 (1.06–1.15)^a^
Female gender	0.74 (0.67–0.81)^a^
Care Dependency Scale (CDS) ≥ 70 completely independent	Reference
≥ 60–69 to a great extent independent	3.20 (2.74–3.72)^a^
≥ 45–59 partially dependent	8.83 (7.59–10.28)^a^
≥ 25–44 to a great extent dependent	23.81 (20.17–28.10)^a^
≤ 24 completely dependent	39.74 (32.72–48.26)^a^
Mental and behavioural disorders	2.31 (2.09–2.56)^a^
Factors influencing health status and contact with health services	1.42 (1.22–1.65)^a^
Diseases of the genitourinary system	0.90 (0.81–1.00)
Diseases of the digestive system	0.85 (0.76–0.95)^a^
Diseases of the musculoskeletal system and connective tissue	0.78 (0.70–0.86)^a^

*AIC* Akaike information criterion, *ICC* intraclass correlation coefficient, *OR* odds ratio, *95% CI* 95% confidence interval, *MOR* median odds ratio, *80% IOR* 80% interval odds ratio

^a^statistically significant based on the 95%CI

With regard to the organisational factors (specific contextual effects), the availability of guidelines regarding restraints (OR 0.60, 95% CI 0.49–0.74, 80% IOR 0.04–9.30) and refresher courses for at least 80% of ward nursing staff (OR 0.75, CI 0.64–0.89, 80% IOR 0.05–11.64) were associated with less restraint use. The availability of a multi-disciplinary expert committee and regular audits to ensure compliance with the protocol/guidelines regarding restraints were not selected for the model, indicating that these factors are not relevant concerning restraint use, from a statistical point of view. Also, the variable country was not selected for the model, despite large descriptive differences in prevalence rates.

Only considering the fixed effects (patient characteristics and organisational factors), the model could explain 24% of the variance in restraint use (marginal *R*^2^ = 0.24). By adding the random effect (hospital as cluster variable), the model explains 55% of the variance in restraint use (conditional *R*^2^ = 0.55). The log-likelihood ratio test was statistically significant (*p*-value < 0.000), indicating that adding hospital as a random effect (cluster) does improve the model. Additionally, the ICC (0.41) shows that the random effect is also relevant from a clinical point of view. This means that a relevant part of the variance in restraint use can be explained at the hospital level. The MOR (4.22) also highlights that there is rather large heterogeneity between hospitals.

## Discussion

In this secondary data analysis of cross-sectional data on restraint use in Swiss and Austrian hospitals, we analysed the impact of organisational factors (specific contextual effects) on the use of restraints in the somatic acute care hospital setting, as well as whether a hospital general effect exists. Overall, the restraint prevalence rate was 8.7%. We found that the availability of guidelines regarding restraint use on the institutional level and refresher courses for at least 80% of ward nursing staff in the last 2 years are associated with less restraint use. However, the wide 80% IORs put the impact of these organisational factors in perspective. No association was found for the availability of a multi-disciplinary expert committee regarding restraint use within the institution and regular audits on the ward level to ensure compliance with the guidelines regarding restraint use. Furthermore, the findings show that a relevant part of the variance in restraint use is explained at the hospital level (random effect), suggesting that a hospital general effect exists regarding restraint use. The difference between hospitals also appears to be greater than that between countries, as might have been expected given the much higher restraint prevalence rate in Switzerland (the country variable was not selected for the model). Thus, there is evidence that, in similar patient situations, restraints are used more frequently in some hospitals than in others (up to 4 times). This finding supports assumptions from the literature that, regarding restraint use, local habits, routine and institutional culture seem to play an important role [18–21, 44]. Such routine or habitual restraint use, independent of an objective and evidence-based evaluation, violates professional values and fundamental human rights. Therefore, critical interprofessional reflections on the current restrictive practice within hospitals are needed to minimise non-professional, non-legal and non-ethical restraint use. However, based on well-known safety models, like the Swiss cheese model, we know that patient safety is not only influenced by health professionals involved in direct patient care (micro level) [45]. The conditions within an institution (meso level) and on a national level (macro level) also have a significant impact on patient safety. For this reason, critical reflection on current restraint practices should take place on micro, meso and macro level.

On the micro level, a critical interprofessional reflection of practice is only possible with appropriate knowledge about the topic of interest. Regarding restraint use, it is widely discussed that health professionals in the hospital setting do not have sufficient knowledge and expertise [21]. As a result, restraints are often applied in situations that are not appropriate [14, 19, 22, 46]. For example, restraints are used for fall prevention, even though there is growing evidence that restraints are ineffective in preventing falls [16, 17]. Also, in this study, indications could be found that knowledge influences the use of restraints, since attending a refresher course is associated with less restraint use. Thus, in line with the recommendations of a review related to a Cochrane protocol regarding restraint reduction in general hospitals [47, 48], education of health professionals seems to be a relevant component for restraint reduction. In this regard, it seems important that an interprofessional approach is taken, as this is the only way to change the institutional culture, the perception of risk-taking and the work ethic [44]. In particular, the results of this study show how important these institution-specific aspects seem to be (hospital general effect).

However, changes in these institution-specific aspects also require a strong commitment from the meso level. First of all, there is a need for open discussion within an institution, for example to clarify responsibilities for safety [44]. Especially in the care of elderly people, the assessment of security issues needs different perspectives [49]. For example, functional needs must also be weighed in the decision-making process in terms of using or not using restraints. This is even more important as, like the findings show, older and more care-dependent patients have an increased risk of being restrained during their hospital stay, and as restraint use is associated with functional decline [1]. In addition, mental and behavioural disorders are associated with a higher use of restraints. This means that a very vulnerable patient group is most affected, i.e. patients who often cannot stand up for themselves; therefore, ethical considerations are even more important. In this regard, the management has the responsibility to support front-line staff by influencing the structural conditions for example, as also shown in this study, by providing policies/ guidelines that support decision-making or at least restraint management in line with legal and ethical requirements [18, 20–22, 24, 47]. In addition, they can adapt the infrastructural conditions, for example by removing restraint equipment from the wards, as it is known that the availability of restraint equipment influences its use [23]. It seems interesting that, in this study, regular audits and the availability of an expert committee were not found to be associated with restraint use. A possible explanation might be that, for both tasks, the individual person (who conducts the audit or is a member of the expert committee) must be able to critically reflect on the situation in which restraints are used and, in particular, to take an outsider perspective in order to identify restraint use due to the institutional culture or attitudes. However, as discussed above, the knowledge and expertise of the individual person might be insufficient and therefore no effect of these two organisational factors could be measured.

To support critical reflection on the micro and meso level and thus to support the change in restrictive practice in order to protect human rights of personal freedom and to ensure professional restraint use, interventions should also be taken on the national (macro) level [45, 50, 51]. For example, in both included countries (Switzerland and Austria), clear legal regulations regarding restraint use in the hospital setting are lacking [15]. However, clear regulations, professional statements of nurses or medical associations and national guidelines would help institutions to clarify their policies, would support the uniform education of health professionals and would provide a basis for national quality improvement programs in the hospital setting. Such programs often lead to more uniform monitoring of restraint use within institutions and thus enable comparison, which are both important aspects in restraint reduction [24, 52].

As restraint use is a very sensitive issue, in this respect, a national quality measurement with a risk-adjusted comparison should be considered. This is the only way to guarantee that the different patient mix of institutions is taken into account and that a fair statistical comparison can be made [45]. Moreover, there is otherwise a risk that institutions with a higher restraint prevalence rate will only see their patient mix (e.g. older, more care-dependent) as the reason for the higher rate and will then reflect on the institution-specific aspects insufficiently. However, as described, this critical reflection seems to be essential for less restrictive practice. In addition, such efforts on the national level could stimulate a more open information policy regarding restraint use in hospitals, more critical thinking about restrictive practice in general and open discussions both within institutions but also in society. These aspects are well-known from similar approaches in the mental health or long-term care setting [53, 54].

### Limitations

Beside its relevant findings, this study has some limitations. Firstly, there are limitations related to the LPZ 2.0 instrument. Some organisational factors expected to be associated with restraint use (e.g. nurse to patient ratio) and health professional-related factors were not assessed with LPZ 2.0. It is, therefore, possible that the impact of the included organisational factors (specific contextual effects) is over- or underestimated as is the relevance of the hospital general effect. Also, the ward level could not be included in the models, since using the LPZ 2.0 instrument restraint use is assessed over a 30 day period in the corresponding hospital without taking into account on which ward the restraint was used (current ward or other). This seems particularly relevant to us, as previous evidence suggests that there are differences in restraint use depending on ward (type) [55]. Thus, future studies should address the inclusion of the ward level. Additionally, data on medical diagnoses are not satisfactorily collected within the LPZ 2.0 instrument. From a statistical point of view, the assessment of ICD-10 diagnosis groups instead of specific ICD-10 diagnosis codes may lead to an over- or underestimation of each diagnosis group. In addition, clinical interpretation and implication is hampered by these very broad and imprecise groups.

Secondly, it is possible that a selection bias exists. Patients who could not give informed consent and had no legal representative available had to be excluded. It could be that these patients were at high risk for restraint use and, therefore, the prevalence rate might be underestimated. Also, the impact of the predictors might be slightly different when including these patients in the analysis. Similar consequences could also be caused by a potential recall or documentation bias because restraint use was assessed over a period of 30 days. However, it is known that, regarding restraint use, the documentation is often incomplete [5, 15]. Thirdly, the cross-sectional design has its limitations; on the one hand, the patient situations under investigation can fluctuate strongly within institutions on the measurement day and, on the other hand, no causal correlations can be identified using a cross-section design. For example, greater care dependency could lead to restraint use, but could also be a consequence of restraint use. Fourth, possibilities and limitations of different methodological approaches for variable selection are controversially discussed as well as for our chosen approach using AIC selection [56]. With our approach there is a risk that variables are incorrectly excluded from the model (false negatives). However, in comparison to the full model (Additional File 2) our results with variable selection (Table 3) differs only slightly. In terms of an exploratory design, the AIC approach seemed to us to be a useful way of obtaining an initial overview of the topic, reduced in complexity. Nevertheless, in order to improve modelling possibilities/strategies and to obtain more comparable and robust results in general, intensified research attention on restraint use in the hospital setting must be established.

Despite these limitations, the results are expected to be generalisable due to the large sample of two countries using the same data collection method. They provide important indications for future quality development efforts. In this context, it seems to be of interest to investigate explanations for the additional 31% of explained variance on the hospital level (hospital general effect). The inclusion of further structural characteristics in data collection and a subsequent analysis or a qualitative approach, for example by observing the (interprofessional) processes surrounding restraint use, could be helpful in this regard.

## Conclusions

Regarding restraint use, a hospital general effect exists. This indicates that restraints are used more frequently in certain hospitals than in others, even when considering the different patient mix. To provide restraint-free care as much as possible requires both specific knowledge and appropriate structures. Based on these findings, considerable potential for restraint reduction appears to exist in the interprofessional critical reflection of decision-making processes within a hospital; especially, the identification of situations in which restraints were used out of routine or institutional culture. This critical reflection ideally goes along with addressing the knowledge and attitudes towards restraints of the interprofessional team as well as of the management. A clear national (legal) regulation regarding restraint use could support a change in practice.

## Supplementary Information

**Additional file 1.** R Codes of the multilevel model. The R codes used for the multilevel model are provided for transparency.

**Additional file 2.** Multilevel full model. A multilevel full model, including all possible fixed effects, is provided.

## Data Availability

The data that support the findings of this study are available from Swiss National Association for Quality Development in Hospitals and Clinics (Swiss data) and Department of Nursing Science from the Medical University of Graz (Austrian data) but restrictions apply to the availability of these data, which were used under license for the current study, and so are not publicly available.

## Abbreviations

LPZ: Landelijke Prevalentiemeting Zorgkwaliteit
ICD-10: International Statistical Classification of Diseases and Related Health Problems 10^th^ Revision
CDS: Care Dependency Scale
95% CI: 95% confidence intervals
IQR: Interquartile range
AIC: Akaike information criterion
ICC: Intraclass correlation coefficient
VIF: Variance inflation factor
OR: Odds ratio
MOR: median odds ratio
80% IOR: 80% interval odds ratio
