# Supplementary material for: Variation in restraint use between hospitals: a multilevel analysis of multicentre prevalence measurements in Switzerland and Austria
Source: BMC Health Serv Res. 2021 Apr 20;21:367. doi: 10.1186/s12913-021-06362-y (PMC8056521; doi:10.1186/s12913-021-06362-y)
Supplement: Supplementary file 1 — Additional file 1. R Codes of the multilevel model. The R codes used for the multilevel model are provided for transparency. [file 12913_2021_6362_MOESM1_ESM.pdf]

Additional file 1: R Codes of the multilevel model

Below the R codes used for the multilevel model are provided for transparency (1. variable selection and 2. multilevel modeling).

<u>1. Variable selection on two subsets from a random split of the data</u>

````
library(tidyverse)
library(Hmisc)
library("jtools")
library(MASS)
```
sample splitting
```{r first and second random data subset}

set.seed(20200124)

#generate first and second random data subset

CH_AT <- CH_AT %>% mutate(id = row_number())
head(CH_AT$id)

first <- CH_AT %>% sample_frac(.50)

second  <- anti_join(CH_AT, first, by = 'id')

#Check proportion outcome

tab2=table(first$Rest_Prev)
prop.table(tab2)

tab2=table(second$Rest_Prev)
prop.table(tab2)
```
first data subset: logistic regression keep IDresponsible
```{r first}

glm_first_all_s <- glm(Rest_Prev ~ ProjectID + Age_stand + Age_quadr + G_gender
+ Length_stand + G_pat_surgery + BFH_PAS_Kat + BFH_diag_infect +
BFH_diag_canc + BFH_diag_blood + BFH_diag_endo + BFH_diag_psych +
BFH_diag_nerve  + BFH_diag_eye + BFH_diag_ear + BFH_diag_cardio +
BFH_diag_lung + BFH_diag_digest + BFH_diag_skin + BFH_diag_motor +
BFH_diag_urogen + BFH_diag_other  + BFH_diag_accident + BFH_diag_external +
BFH_diag_influence + InstitutionForm_QI_Inst_Rest_1 +
InstitutionForm_QI_Inst_Rest_2 + QI_Ward_Rest_1 + QI_Ward_Rest_4 +
IDresponsible, family = binomial, data=first)
#summary(glm_first_all_s)
#summ(glm_first_all_s, vifs = TRUE)
#exp(coef (glm_first_all_s))
#exp(confint (glm_first_all_s))
````

#AIC backwards procedure

glm_first_all_s_step <- glm_first_all_s %>% stepAIC(scope = list(lower = ~IDresponsible), trace = FALSE)
summ(glm_first_all_s_step, vifs = TRUE)
#exp(coef (glm_first_all_s_step))
#exp(confint (glm_first_all_s_step))
```
second data subset: logistic regression keep IDresponsible
```{r second}

glm_second_all_s <- glm(Rest_Prev ~ ProjectID + Age_stand + Age_quadr + G_gender + Length_stand + G_pat_surgery + BFH_PAS_Kat + BFH_diag_infect + BFH_diag_canc + BFH_diag_blood + BFH_diag_endo + BFH_diag_psych + BFH_diag_nerve  + BFH_diag_eye + BFH_diag_ear + BFH_diag_cardio + BFH_diag_lung + BFH_diag_digest + BFH_diag_skin + BFH_diag_motor + BFH_diag_urogen + BFH_diag_other  + BFH_diag_accident + BFH_diag_external + BFH_diag_influence + InstitutionForm_QI_Inst_Rest_1 + InstitutionForm_QI_Inst_Rest_2 + QI_Ward_Rest_1 + QI_Ward_Rest_4 + IDresponsible, family = binomial, data=second)
#summary(glm_second_all_s)
#summ(glm_second_all_s, vifs = TRUE)
#exp(coef (glm_second_all_s))
#exp(confint (glm_second_all_s))

# AIC backwards procedure

glm_second_all_s_step <- glm_second_all_s %>% stepAIC(scope = list(lower = ~IDresponsible), trace = FALSE)
summ(glm_second_all_s_step, vifs = TRUE)
#exp(coef (glm_second_all_s_step))
#exp(confint (glm_second_all_s_step))
```

<u>2. Multilevel model with variables included in both selections</u>

library("tidyverse")
library("Hmisc")
library("lme4")
library("jtools")
library("MuMIn")
```
Interceptonly model
```{r}
interceptonly<-glmer(Rest_Prev ~ 1 + (1|IDresponsible), family = binomial, data=CH_AT)
summary(interceptonly)
confint(interceptonly)
```

```
Multilevel model full dataset
```{r}

final_all<-glmer(Rest_Prev ~ poly(Age_stand, degree=2, raw=T) + G_gender +
BFH_PAS_Kat + BFH_diag_psych + BFH_diag_digest + BFH_diag_motor +
BFH_diag_urogen + BFH_diag_influence + InstitutionForm_QI_Inst_Rest_1  +
QI_Ward_Rest_4  + (1 | IDresponsible), family= binomial, data=CH_AT, control =
glmerControl(optimizer = "optimx", optCtrl = list(method = "nlminb")))
summary(final_all)
se <- sqrt(diag(vcov(final_all)))
(tab <- cbind(Est = fixef(final_all), LL = fixef(final_all) - 1.96 * se, UL = fixef(final_all) +
1.96 *
se))
exp(tab)
```
Explained variance Multilevel model and intercept only
```{r}
r.squaredGLMM(final_all)
r.squaredGLMM(interceptonly)
```
check for significance of random effect
```{r}
# H0 model without random effect
m0 <- glm(Rest_Prev ~ poly(Age_stand, degree=2, raw=T) + G_gender +
BFH_PAS_Kat + BFH_diag_psych + BFH_diag_digest + BFH_diag_motor +
BFH_diag_urogen + BFH_diag_influence + InstitutionForm_QI_Inst_Rest_1  +
QI_Ward_Rest_4, family= binomial, data=CH_AT)
logLik(m0)

## model with random effect
m1 <- final_all
logLik(m1)

## log-Likelihood ratio test
t <- as.numeric(2 * (logLik(m1) - logLik(m0)))
df <- as.numeric((attr(logLik(m1), "df") - attr(logLik(m0), "df")))
pval <- 2 * (1 - pchisq(q = t, df = df))
pval

```
ICC
```{r}
library(sjPlot)

tab_model(final_all, show.df = TRUE)
```
```
