# Supplementary material for: Variation in restraint use between hospitals: a multilevel analysis of multicentre prevalence measurements in Switzerland and Austria
Source: BMC Health Serv Res. 2021 Apr 20;21:367. doi: 10.1186/s12913-021-06362-y (PMC8056521; doi:10.1186/s12913-021-06362-y)
Supplement: Supplementary file 2 — Additional file 2. Multilevel full model. A multilevel full model, including all possible fixed effects, is provided. [file 12913_2021_6362_MOESM2_ESM.pdf]

Additional file 2: Multilevel full model

A multilevel full model, including all possible fixed effects, is provided.

Table A: Multilevel logistic regression full model

**Model:** AIC 12564.0; marginal R^2=0.29; conditional R^2=0.57; ICC=0.39, MOR=3.98	
**Random effect**	**Variance (SD)**
Hospital (intercept)	2.09 (1.45)
**Fixed effects**	**OR (95% CI)**
(intercept)	0.01 (0.01-0.02)*
Country<br>Austria	Reference
Switzerland	2.70 (1.54-4.75)*
Organisational factors (specific contextual effects)	
Guidelines regarding restraint (yes)	0.60 (0.49-0.75)*<br>80% IOR: (0.04-8.33)
Multi-disciplinary expert committee (yes)	0.93 (0.79-1.09)<br>80% IOR: (0.07-12.80)
Regular audits (yes)	1.15 (0.98-1.34)<br>80% IOR: (0.08-15.79)
Refresher course regarding restraints (yes)	0.77 (0.65-0.90)*<br>80% IOR: (0.06-10.53)
Patient characteristics	
Age in years (1st degree)	1.20 (1.12-1.27)*
Age in years squared (2nd degree)	1.10 (1.06-1.15)*
Number of days since admission to hospital	1.03 (0.99-1.07)
Female gender	0.74 (0.67-0.81)*
Surgical intervention in the two weeks prior to data collection (yes)	1.04 (0.94-1.16)
Care Dependency Scale (CDS)<br>≥ 70 completely independent	Reference
≥ 60-69 to a great extent independent	3.11 (2.67-3.63)*
≥ 45-59 partially dependent	8.56 (7.34-9.98)*
≥ 25-44 to a great extent dependent	22.96 (19.40-27.17)*
≤ 24 completely dependent	37.79 (31.00-46.07)*
Mental and behavioural disorders	2.29 (2.06-2.54)*
Factors influencing health status and contact with health services	1.36 (1.17-1.59)*
External causes of morbidity and mortality	1.35 (1.02-1.78)*
Diseases of the eye and adnexa	1.22 (1.02-1.46)*
Symptoms, signs and abnormal clinical and laboratory findings, not elsewhere classified	1.15 (0.95-1.39)

Diseases of the ear and mastoid process	1.09 (0.83-1.45)
Injury, poisoning and certain other consequences of external causes	1.09 (0.92-1.30)
Diseases of the nervous system	1.09 (0.96-1.23)
Diseases of the blood and blood-forming organs and certain disorders involving the immune mechanism	1.08 (0.96-1.22)
Diseases of the circulatory system	1.06 (0.94-1.19)
Diseases of the respiratory system	1.05 (0.94-1.17)
Certain infectious and parasitic diseases	0.97 (0.85-1.11)
Neoplasms	0.97 (0.86-1.09)
Endocrine, nutritional and metabolic diseases	0.94 (0.85-1.04)
Diseases of the skin and subcutaneous tissue	0.89 (0.75-1.05)
Diseases of the genitourinary system	0.89 (0.80-0.99)*
Diseases of the digestive system	0.85 (0.76-0.95)*
Diseases of the musculoskeletal system and connective tissue	0.77 (0.69-0.85)*
*statistically significant based on the 95%CI	
AIC= Akaike information criterion, ICC=intraclass correlation coefficient, OR=odds ratio, 95% CI=95% confidence interval, MOR=median odds ratio, 80% IOR=80% interval odds ratio	
